# The Insect Growth Regulator Pyriproxyfen Terminates Egg Diapause in the Asian Tiger Mosquito, *Aedes albopictus*

**DOI:** 10.1371/journal.pone.0130499

**Published:** 2015-06-19

**Authors:** Devi S. Suman, Yi Wang, Randy Gaugler

**Affiliations:** Center for Vector Biology, Rutgers University, New Brunswick, NJ, United States of America; New Mexico State University, UNITED STATES

## Abstract

The Asian tiger mosquito, *Aedes albopictus*, is a highly invasive mosquito species that transmits chikungunya and dengue. This species overwinters as diapausing eggs in temperate climates. Early diapause termination may be a beneficial strategy for winter mosquito control; however, a mechanism to terminate the diapause process using chemicals is not known. We tested the hypothesis that a hormonal imbalance caused by the administration of juvenile hormone analog would terminate egg diapause in *A*. *albopictus*. We tested the insect growth regulator pyriproxyfen on all developmental stages to identify a susceptible stage for diapause termination. We found that pyriproxyfen treatment of mosquito eggs terminated embryonic diapause. The highest rates of diapause termination were recorded in newly deposited (78.9%) and fully embryonated (74.7%) eggs at 0.1 and 1 ppm, respectively. Hatching was completed earlier in newly deposited eggs (25–30 days) compared to fully embryonated eggs (71–80 days). The combined mortality from premature diapause termination and ovicidal activity was 98.2% in newly deposited and >98.9% in fully embryonated eggs at 1 ppm. The control diapause eggs did not hatch under diapausing conditions. Pyriproxyfen exposure to larvae, pupae and adults did not prevent the females from ovipositing diapausing eggs. There was no effect of pyriproxyfen on diapausing egg embryonic developmental time. We also observed mortality in diapausing eggs laid by females exposed to pyriproxyfen immediately after blood feeding. There was no mortality in eggs laid by females that survived larval and pupal exposures. In conclusion, diapausing eggs were the more susceptible to pyriproxyfen diapause termination compared to other life stages. This is the first report of diapause termination in *A*. *albopictus *with a juvenile hormone analog. We believe our findings will be useful in developing a new control strategy against overwintering mosquito populations.

## Introduction

The Asian tiger mosquito, *Aedes albopictus*, is one of the world’s worst invasive species [1]. This mosquito is prevalent worldwide and transmits chikungunya and dengue viruses in Asian, African and European countries [2–4]. The Asian tiger mosquito was first discovered in the United States in Houston, Texas in 1985 [5]. In the last 20 years, this mosquito has spread to at least 30 additional states, and is continuously expanding its range [6,7]. One factor contributing to *A*. *albopictus* establishment into new areas is a specialized egg with a thick eggshell that is desiccation tolerant [8,9] and ability to enter diapause [10]. Diapause eggs are large in size and high in lipid content [11], and are more desiccation tolerant than non-diapause eggs [12,13]. The ability of diapause eggs to survive harsh temperate conditions may have facilitated *A*. *albopictus* range expansion [14–16].

There is immense diversity in insect diapause mechanisms, including various life stages. For instance, diapause occurs in eggs in *Bombyx mori*, larvae in *Ostrinia nubilalis*, pupae in *Sarcophaga crassipalpis* and adults in *Culex pipiens* [17,18]. *Aedes albopictus* enters diapause in the pharate larval stage; that is, when the embryo is fully developed. Embryonic diapause is common among insects; for instance, the locusts *Locusta migratoria* and *Oxya yezoensis* enter diapause at the mid-stage of embryogenesis prior to blastokinesis which is quite different from *A*. *albopictus* [19,20]. The mechanism(s) underlying diapause in *A*. *albopictus* is not well understood [11–13, 21,22].

Diapause termination normally occurs when suitable environmental conditions induce physiological and developmental processes [20,23]. Alternatively, insects may terminate diapause when exposed to specific chemicals [20]. Examples of this include a synthetic juvenile hormone for adult *C*. *pipiens* [18], the solvent hexane for flesh fly pupae of *S*. *crassipalpis* [24], a diapause hormone for pupae of *Helicoverpa zea* [25], and the juvenile hormone analogue methoprene and the ecdysteroid hormone 20-hydroxyecdysone for early embryonic eggs of *L*. *migratoria and O*. *yezoensis* [19]. There are no reports on diapause termination in *A*. *albopictus* using a chemical stimulus.

Hormones play a vital role in embryonic development, as well as diapause regulation [20,26]. Previously, we have shown that the juvenile hormone analog pyriproxyfen disrupts embryonic development in non-diapause eggs of *A*. *albopictus*, resulting in ovicidal activity [16]. Ecdysteroid signaling has been shown to regulate embryonic diapause in *A*. *albopictus* and other mosquitoes [21,27]. Ecdysteroid and juvenile hormones together regulate insect molting, metamorphosis and reproduction [22], yet there is no report on juvenile hormone involvement in *A*. *albopictus* diapause. We hypothesize that disruption of the hormonal balance in *A*. *albopictus* resulting from pyriproxyfen exposure would terminate diapause. To identify a susceptible stage for diapause termination, we tested the effect of pyriproxyfen on all life stages including egg (newly deposited and fully embryonated), larvae, pupae and adults.

## Materials and Method

### Mosquito rearing and diapause induction

*Aedes albopictus* eggs from a laboratory colony maintained under non-diapausing conditions (26 ± 2°C, 65% RH and 16L:8D photoperiod) [28], were hatched in dechlorinated tap water in 2L enamel trays. Larvae were reared to the 3^rd^ instar. Dried brewer’s yeast was provided as larval food (30 mg/L) twice a week. To induce diapause, 3^rd^ instar larvae were transferred to a 21°C incubator maintained at short day conditions (8L:16D photoperiod) which are well established as diapause-inducing conditions [14]. Sugar solution (10%) in cotton wicks was provided to adults and guinea pigs were offered for blood feeding.

### Ethics Statement

The study protocol was approved by the Institutional Animal Care and Use Committee Review and Approval (IACUC), Rutgers the State University of New Jersey. The study was performed in strict accordance with the recommendations of IACUC Animal Use Protocol # 86–129 for the care of guinea pig in mosquito research.

### Pyriproxyfen

An emulsifiable concentrate formulation of pyriproxyfen (NyGuard EC10, MGK, Minneapolis, MN, USA) was used to prepare a 1% stock solution and serial dilutions (0.01, 0.1, 0.5 and 1 ppm) for use with eggs, larvae and pupae. All dilutions were prepared in dechlorinated tap water. For adults, a corn oil based formulation was prepared with pyriproxyfen (technical grade 99%, MGK, Minneapolis, MN, USA). A Whatman #1 filter paper (9 x 8 cm) was treated with the oil formulation at 10 mg a.i. pyriproxyfen/m^2^ and dried overnight at room temperature (25 ± 1°C). The treated paper was inserted into a chamber constructed from a 50 mL centrifuge plastic tube blocked at one end with mosquito-proof mesh for aeration. For the control, similar paper was treated with same amount of pyriproxyfen-free corn oil. Treated papers and treatment chambers were used for one repeat and were then discarded.

### Determination of a susceptible stage for diapause termination

**Fig 1:**
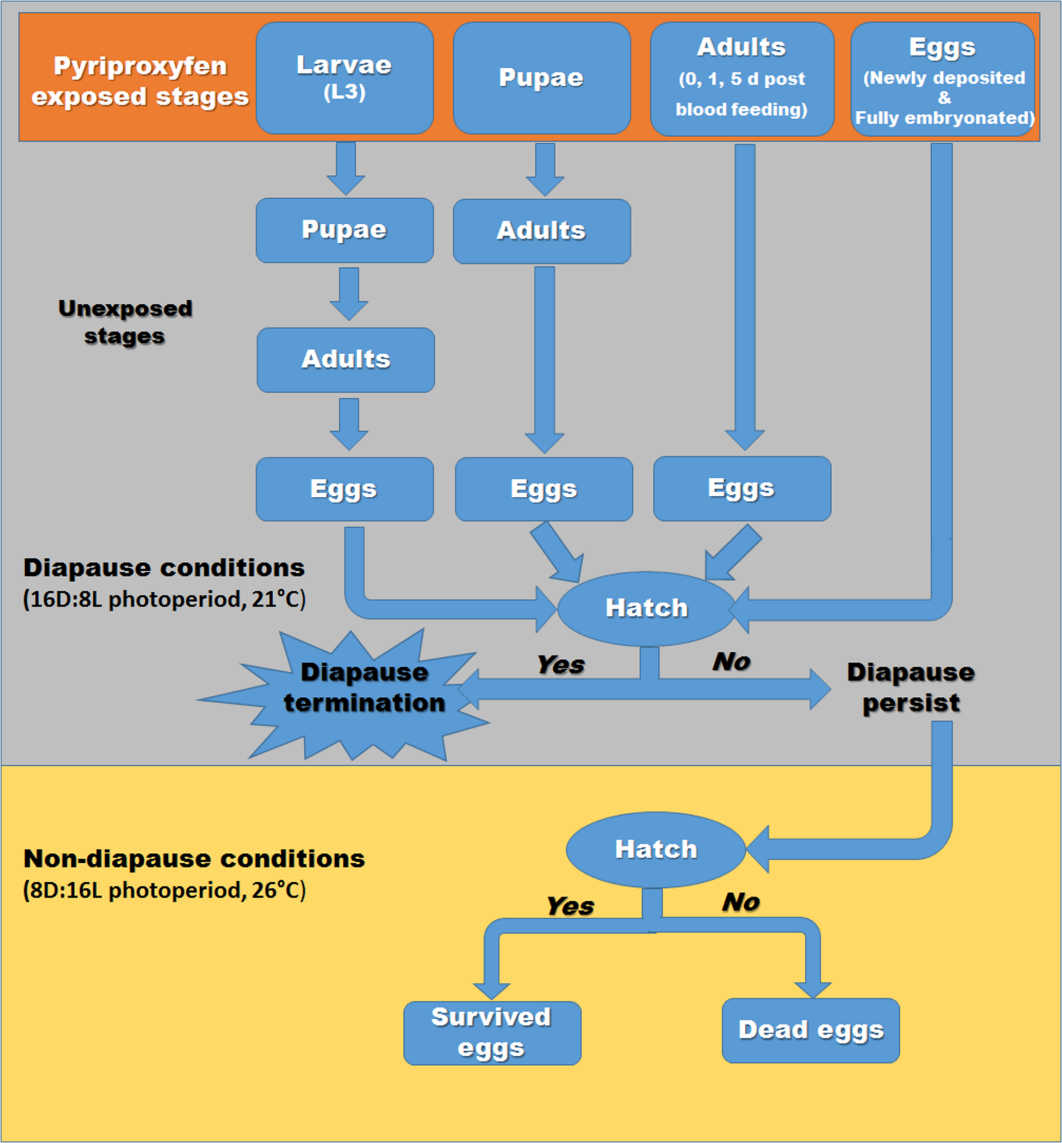
Schematic representation of diapause termination experiment indicating pyriproxyfen exposure of eggs (newly deposited and fully embryonated), larvae, pupae and adult females of *Aedes albopictus* under diapause conditions (8L:16D photoperiod and 21°C) and ovicidal activity under non-diapause conditions.

After exposure of various life stages (eggs, larvae, pupae or adults) to diapausing conditions (8L:16D photoperiod; 21°C), we determined the diapause status of eggs with the use of a hatching experiment [14] as described in Fig 1.

### Eggs

For this experiment, two developmental stages of diapause eggs were tested: newly deposited eggs and fully embryonated eggs.

The newly deposited eggs were obtained by releasing gravid females (5 d post blood-feeding) into an oviposition container to oviposit directly on a treated oviposition paper (n = 6,325) under diapausing conditions. To test newly deposited eggs (immediately laid), we set up oviposition containers treated with 0.01, 0.1 and 1 ppm of pyriproxyfen in 250 mL water. Control container were not treated. Each container received an oviposition paper strip (4 cm strip of Whatman #1 filter paper) encircling the inner container wall. The paper was saturated with treated water. Eggs were counted and kept in the same treated water to observe hatching.

To test fully embryonated eggs (n = 2,579), two-week-old diapausing eggs were exposed to 0.01, 0.1 and 1 ppm of pyriproxyfen concentrations in 250 mL water under diapausing conditions and allowed to hatch. Newly deposited and fully embryonated control eggs were treated with water only. No larval food was added in any treatment or control as a hatching stimulus.

In all experiments, treatments and control of both newly deposited and fully embryonated eggs were in three replicates and repeated three times.

### Larvae and pupae

We exposed 100 3^rd^ instars and recently pupated mosquitoes to each pyriproxyfen concentration (0.01, 0.1, 0.5 and 1 ppm) in 500 mL water under diapause conditions. In controls, larvae and pupae were not treated with any chemical. Larvae were provisioned with dried Brewer’s yeast (30 mg/L). Exposure was terminated when the larvae or pupae molted to the subsequent stage. Surviving pupae and adults from the respective treatments were collected and transferred into untreated adult cages (30 x 30 x 30 cm). Adults were provided 10% sugar solution *ad libitum* and were blood fed on guinea pig one week after emergence. Oviposition containers with untreated water (250 mL) were provided for egg laying. Eggs (n = 4,680 in larval exposure and n = 3,801 in pupal exposure) were counted and allowed to hatch under diapausing conditions for one month. To measure diapause termination, we determined the hatch rate of eggs laid by adults exposed during larval and pupal stages to pyriproxyfen. All experiments consists of three replicates and were repeated three times.

### Adults

Adult mosquitoes (7–10 d old) reared under diapausing conditions were blood fed before being separated into four groups of 20. Each group was exposed for 10 min to a corn oil formulation (10 mg pyriproxyfen/m^2^) on Whatman # 1 filter paper (72 cm^2^) at a specific time interval post-blood feeding (1 h, 1 and 5 d). Exposed mosquitoes were held in a 1 liter container with 200 mL untreated water for 2 d, allowing for oviposition. Eggs (n = 4,335) were counted and observed for hatching under diapausing conditions for 30 days. Controls were exposed to only corn oil after blood feeding. Experiments were set up in three replicates and repeated three times.

### Diapause termination

Eggs in diapause are not expected hatch under diapausing conditions (8L:16D photoperiod and 21°C); therefore, if we observed eggs hatching under these conditions we considered them to have experienced diapause termination. The diapause eggs from different treatment groups (eggs, larvae, pupae and adults) and controls were observed for hatching every other day under diapausing conditions. All larvae were counted and removed during each observation. Diapause termination was calculated after there was no hatch observed for 14 consecutive days.

### Ovicidal activity

Under non-diapausing conditions (26 ± 2°C and 16L:8D photoperiod), observations of diapause egg hatch of all treatment groups was extended (Fig 1). We observed egg hatch on alternate days, and first instars were counted and removed. Eggs that remained unhatched after exposure to both diapausing and non-diapausing conditions were considered dead due to ovicidal activity [9].

### Effect of pyriproxyfen on embryonic development

We considered that pyriproxyfen might alter the developmental time of diapause eggs. To examine this, diapause eggs of the same age from both treatment (1 ppm) and controls were observed daily from 0–9 d post-oviposition. Eggs (80–100 per day) were bleached to make the eggshell transparent and photographed to compare the stages for germ band formation, embryo with segmented body, development of thoracic and abdominal extremities such as head capsule, eye, and abdominal segments [9]. Non-diapause eggs were also bleached and observed in the same way as a positive control. Photographs were taken to record embryonic development.

### Statistical analysis

For the statistical analysis, data from all nine replicates were pooled together for each variable and were analyzed for normal distribution and equal variance test. Diapause termination and ovicidal activity in both newly deposited and fully embryonated diapause eggs were calculated from recorded egg hatch as described above. In larval, pupal and adult exposures, diapause termination were measured by egg hatching experiments under diapausing conditions and ovicidal activity on eggs under non-diapausing conditions. One-way Analysis of Variance (ANOVA) was performed for significance analysis of all pyriproxyfen treatments and controls using the statistical package PASW statistics 18 (SPSS Inc.). Multiple range test was used to compare the means of eggs, larval, pupal and adult exposures and their respective controls for significant differences using Fisher’s least significant difference (LSD) at *P <* 0.05. Statistical results were presented with degree of freedoms (df) between and within groups, F-ratios and P-values. Data is presented as mean ± standard error.

## Results

### Diapause termination with pyriproxyfen exposure to eggs

**Fig 2:**
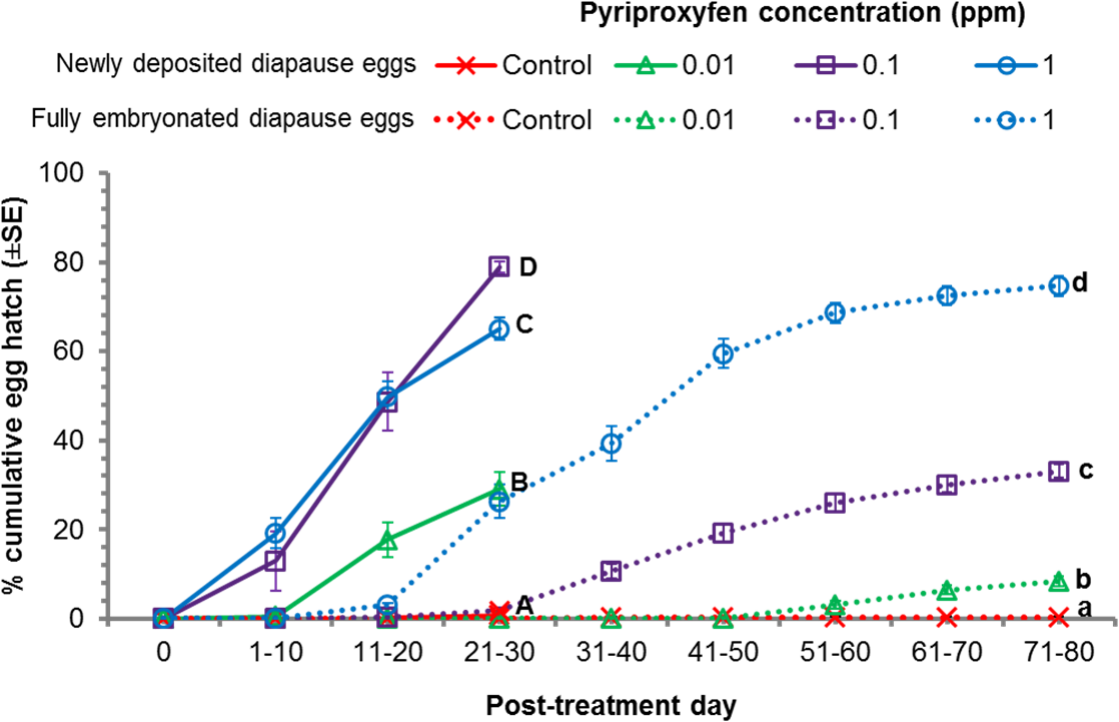
Hatching of newly deposited and fully embryonated diapause eggs of *Aedes albopictus* under diapausing conditions (8L:16D and 21°C) with pyriproxyfen exposure (0.01–1 ppm). Solid lines of newly deposited diapause eggs designated with same capital letters and dotted lines for diapause fully embryonated eggs designated with small letters are not different significantly (One-way ANOVA, p < 0.05).

Newly deposited diapause eggs exhibited a significantly higher hatch rate in pyriproxyfen treatments than the controls (Fig 2, Table 1; df = 3, 32; f = 215.6; *P* = 0.0001). Hatching was lowest at 0.01 ppm (29.1 ± 3.8%) and highest at 0.1 ppm (78.9 ± 1.3%). Diapause egg hatch was 78.9 ± 1.3% at 0.1 ppm which was higher than that of 1ppm (65.0 ± 2.6%). This was because 1 ppm killed more eggs than the 0.1 ppm. Egg mortality was significantly higher at 0.1 (8.4 ± 0.6%) and 1.0 ppm (33.1 ± 2.5%) pyriproxyfen than in controls (2.9 ± 0.6%) (Table 1; df = 3, 32; f = 102.4; *P* = 0.0001). Under non-diapausing conditions, newly deposited diapause control eggs showed 96.1 ± 0.7% hatch, confirming the diapause status of eggs (Table 1; df = 3, 32, f = 512.9, *P* = 0.0001).

**Table 1:** Hatching of newly deposited and fully embryonated diapause eggs of *Aedes albopictus* with exposure of pyriproxyfen under diapausing conditions (8L:16D photoperiod and 21°C) and stimulated non-diapausing conditions (16L:8D photoperiod and 26°C).

	Diapause eggs exposure to pyriproxyfen (ppm)				Statistical significance (One-way ANOVA)
	Parameters (%)	Control	0.01	0.1	1	
**Newly deposited diapause eggs**					
Hatch under diapausing conditions	1.0±0.3^a^	29.1±3.8^b^	78.9±1.3^c^	65.0±2.6^d^	df = 3,32; f = 215.6; *P* = 0.0001
Hatch under non-diapausing conditions	96.1±0.7^a^	66.4±3.6^b^	12.6±1.3^c^	1.8±0.4^d^	df = 3,32; f = 512.9; *P* = 0.0001
Dead eggs	2.9±0.6^a^	4.5±0.8^ab^	8.4±0.6^b^	33.1±2.5^c^	df = 3,32; f = 102.4; *P* = 0.0001
**Fully embryonated diapause eggs**					
Hatch under diapausing conditions	0.2±0.2^a^	8.4±1.2^b^	33.1±1.6^c^	74.7±2.3^d^	df = 3,32; f = 483.3; *P* = 0.0001
Hatch under non-diapausing conditions	98.5±0.4^a^	85.3±1.6^b^	57.2±1.9^c^	1.0±0.4^d^	df = 3, 32; f = 1138.8; *P* = 0.0001
Dead eggs	1.3±0.3^a^	6.3±0.6^b^	9.7±0.7^b^	24.2±2.3 ^c^	df = 3, 32; f = 61.3; *P* = 0.0001

Dead eggs were the percentage of eggs unhatched under both conditions.

All the values are represented in mean ± SE.

Mean values in a row denoted with same letter are not significantly different with One-way ANOVA at p<0.05.

Fully embryonated diapause egg hatch was concentration dependent. Hatch was lowest at 0.01 ppm (8.4 ± 1.2%) and highest at 1.0 ppm of pyriproxyfen (74.7 ± 2.3%), and the hatch rate was significantly higher in all treatments compared to control group (0.22 ± 0.22%) under diapause conditions (Fig 2; df = 3, 32; f = 483.3; *P* = 0.0001). Under non-diapausing stimulated egg hatching conditions, 98.5 ± 0.4% hatch was seen in controls which was higher than the treatments (Table 1; df = 3, 32; f = 1138.8; *P* = 0.0001). Pyriproxyfen caused 6 to 24% mortality in fully embryonated diapause eggs. Mortality rates were concentration dependent (Table 1; df = 3, 32; f = 61.3; *P* = 0.0001).

The hatching period was shorter in the newly deposited diapause eggs compared to fully embryonated ones (Fig 2). For newly deposited diapause eggs, hatch began 5–7 days after treatment and that reached to maximum within 25–30 days. Whereas, for fully embryonated diapause eggs hatch began 10 days after treatment and reached to maximum by 71–80 days of treatment. Hatch durations of both newly deposited and fully embryonated diapause eggs were concentration dependent (Fig 2).

### Effect of larval pyriproxyfen exposure on egg diapause termination

Adult emergence from pyriproxyfen treated larvae was 73.4 ± 2.4% at 0.01 ppb, 70.7 ± 2.6% at 0.1 ppb, and 56.1 ± 3.1% at 1.0 ppb. Controls had 91.6 ± 0.9% adult emergence. Under diapause conditions, eggs laid by females from either treatment or control group failed to hatch confirming diapause status of the eggs. However, under non-diapausing conditions, egg hatch was 84.8 ± 2.0, 83.3 ± 2.8 and 79.1 ± 2.8% for 0.01, 0.1, 0.5 ppb treatment groups and 88.8 ± 2.1% for control groups, respectively. There was no significant difference among the treatments and controls (df = 3, 32; f = 2.68; *P* = 0.063).

### Effect of pupal pyriproxyfen exposure on egg diapause termination

Adult emergence from pupae exposed to pyriproxyfen was 48.7 ± 2.2% at 0.01 ppb, 26.3 ± 1.9% at 0.1 ppb, and 12.1 ± 1.1% at 0.5 ppb and 93.7 ± 0.8% in controls. No diapause termination was observed in eggs laid by females emerged from any pupal treatment since there was no hatch observed under diapausing conditions. Under non-diapausing conditions, hatch was 89.8 ± 1.3% at 0.01 ppb, 89.6 ± 1.1% at 0.1 ppb, 86.3 ± 1.9% at 0.5 ppb treatments and 93.3 ± 1.0% in controls. Hatching was not significantly different among the treatment and control groups except between control and 0.5 ppb pyriproxyfen (df = 3, 32; f = 4.19; *P* = 0.013).

### Effect of adult pyriproxyfen exposure on egg diapause termination

Adult female exposure to pyriproxyfen (10 mg a.i./m^2^) at 1 h, 1 day, and 5 days of post-blood feeding did not interfere with the diapause status of eggs and we did not observe any egg hatch under diapausing conditions. However, eggs laid by females exposed to pyriproxyfen after 1 h of blood feeding had a significantly lower hatch rate (37.1 ± 9.8%) than the control (91.7 ± 1.8%) and 1 and 5 day post-blood feeding treatments (92.3 ± 2.7%, 96.2 ± 1.5%) under stimulated non-diapausing conditions (df = 3, 32; f = 92.17; *P* = 0.0001).

### Effects of pyriproxyfen on diapause egg embryonic development

**Fig 3:**
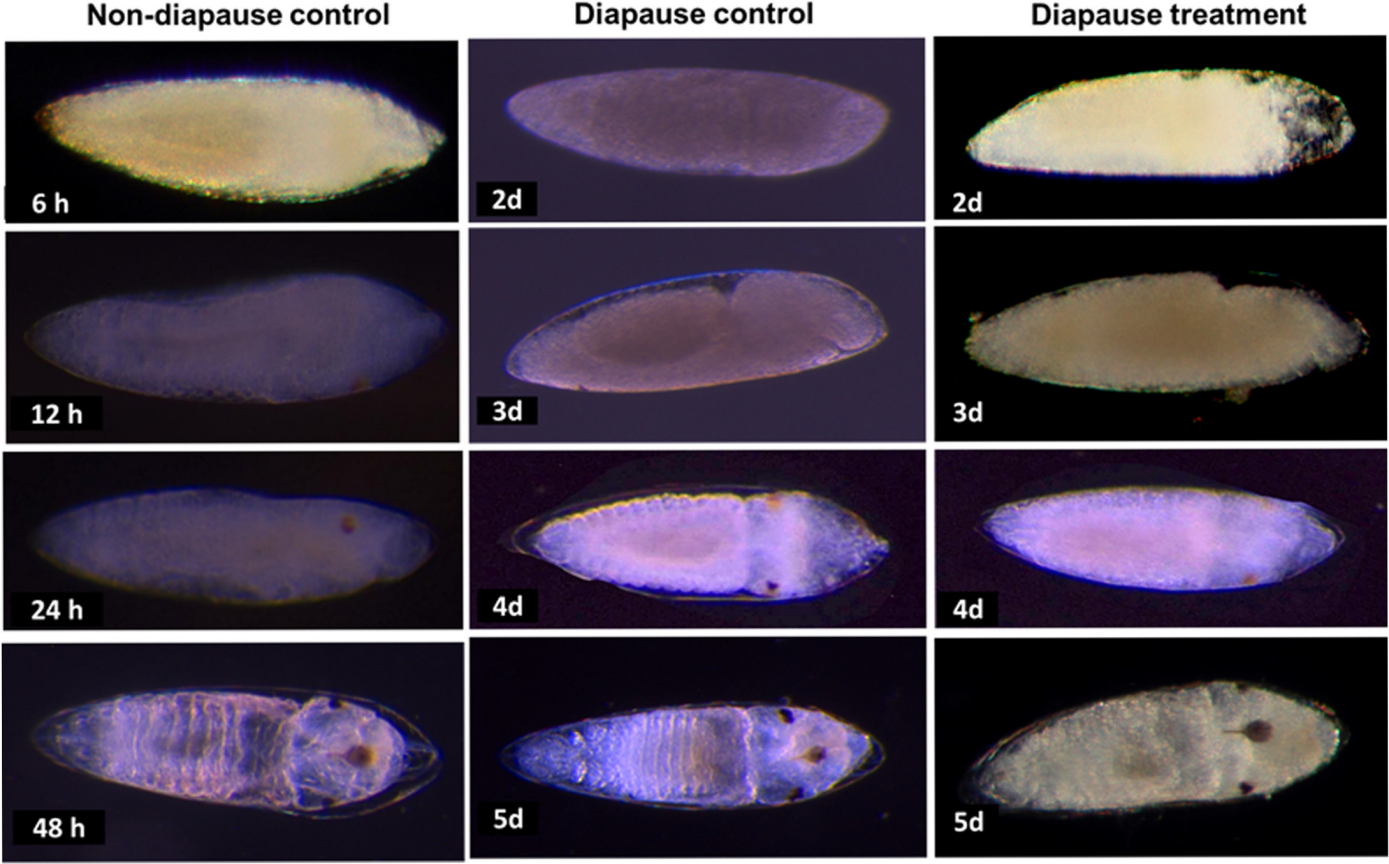
Comparison of developmental stages of non-diapause, diapause control and diapause treatment (1 ppm pyriproxyfen) eggs of *Aedes albopictus*. Non-diapause eggs (16L:8D photoperiod and 26°C) showed 6, 12, 24 and 48 h developmental intervals, whereas, diapause eggs (8L:16D photoperiod and 21°C), both control and treatment showed 2, 3, 4, and 5 d post-oviposition developmental intervals.

Based on morphological observations of embryonic development including germ band formation, embryo with segmented body, and the development of thoracic and abdominal extremities such as eyes, head capsule and abdominal segments, we did not detect differences between pyriproxyfen treated (1 ppm) and untreated newly deposited diapause eggs (Fig 3). Additionally, control and treated diapause eggs showed similar developmental times, with germ band formation in embryos being initiated on the second day after being laid and a fully differentiated embryo completion by day five. In contrast to diapause eggs, non-diapause eggs initiated development few hours after oviposition and completed it within 48 h (Fig 3).

## Discussion

Embryonic diapause is a vital mechanism of overwintering *A*. *albopictus* survival in temperate [14,22]. Diapausing eggs are physiologically and biochemically different from non-diapausing eggs [11–16]. Females lay diapause eggs when their various stages are continuously exposed to short light periods and lower temperature [14]. The lowest hatch rate of control eggs (0–1.0%) under diapausing conditions in all experiments also showed that *A*. *albopictus* mosquitoes produced the complete batch of diapause eggs when reared under 8 h light at 21°C temperature. We found that among all the life stages of *A*. *albopictus*, eggs were the most susceptible stage to juvenile hormone analog for diapause termination. We report, for the first time, egg diapause termination in *A*. *albopictus* caused by pyriproxyfen. Specifically, this was a chemically induced hatch under diapausing conditions.

Previously, we demonstrated that newly deposited eggs are more susceptible to insect growth regulators than fully embryonated eggs since chorionic membranes are more permeable in newly deposited eggs [9]. Pyriproxyfen terminated diapause in 78.9% newly deposited eggs at 0.1 ppm, whereas a ten-fold greater concentration was needed to get a similar result in fully embryonated eggs (74.7% diapause termination at 1 ppm). Pyriproxyfen induced hatch in newly deposited diapause eggs earlier (within a week) and achieved complete hatch in one third of time compared to that taken by fully embryonated diapause eggs under diapausing conditions. We propose that fully embryonated eggs were less responsive to insecticides than newly deposited eggs due to hardening of the chorionic membranes that reduced the permeability of insecticides into the egg. Diapause eggs are rich in lipids and more desiccation tolerant compared to non-diapause eggs [12] which may have further reduced their permeability. Whether permeability played an important role in causing this difference needs further investigation.

Pyriproxyfen possesses higher ovicidal activity against non-diapause eggs of multiple mosquito species [9]. We demonstrated that high concentrations of pyriproxyfen (0.1 and 1 ppm) were significantly ovicidal for both newly deposited and fully embryonated diapause eggs. Previously, we found that pyriproxyfen killed 80% of non-diapausing eggs at 1 ppm [9], which was a higher mortality rate than diapause eggs (33%) at similar concentrations. In diapause eggs, pyriproxyfen primarily induced hatch in a large proportion of eggs and killed the remainder, whereas, there was only ovicidal activity seen in non-diapause eggs. Practically, lower hatch rate would not have any negative impact on overall efficacy as hatched larvae would eventually be killed due to the presence of pyriproxyfen in the treated water. Differences in the ovicidal efficacy of pyriproxyfen for diapause and non-diapause eggs might be attributed to the following factors: 1) the presence of extra fatty acids in the diapause eggshell [12,13]; and 2) lower metabolic rate of diapause eggs than the non-diapause eggs due to lower temperature conditions, but, this needs further exploration.

Specific roles of different life stages in diapause have been reported in various insects (14,20,23). We found that exposure of larvae, pupae and adults to pyriproxyfen did not terminate diapause in *A*. *albopictus*. However, pyriproxyfen caused physiological and developmental alterations in all these life-stages [9,28,29]. In contrast, a novel role of juvenile hormone in the adult *C*. *pipiens* has been reported in which juvenile hormone analog initiates ovarian development in diapause mosquito under diapausing conditions [18]. We do not have evidence whether juvenile hormone is involved directly in *A*. *albopictus* diapause or the effects of pyriproxyfen are recovered in adults after molting. Our results suggest that pyriproxyfen treatments of larvae, pupae and adults might not be an effective strategy to manage diapausing *A*. *albopictus* populations.

Pyriproxyfen sterilizes *Anopheles gambiae* and *Anopheles arabiensis* females [30,31]. We observed the same effect in *A*. *albopictus* females exposed to pyriproxyfen 1 hour after blood feeding; they also laid significantly fewer viable diapause eggs. However, similar effects were not seen in mosquitoes exposed one or more days post-blood feeding. This indicates that pyriproxyfen more effectively disrupts embryonic development when mosquitoes are treated at an early stage of ovarian development.

Hormones play a crucial role in insect embryogenesis [26,32]. Pyriproxyfen induced morphological abnormalities in non-diapause embryos *A*. *albopictus* that led to ovicidal activity [9]. However, we did not notice a difference in the morphology of developing embryos, or a difference in the embryonic development periods when comparing pyriproxyfen treated and untreated diapause eggs. This suggests that pyriproxyfen does not accelerate development to break diapause. Although the role of juvenile hormones in *A*. *albopictus* diapause is unknown, this hormone has been associated with diapause in other insect species. For instance, in *L*. *migratoria*, extracellular signal-regulated kinase (ERK) phosphorylation was activated by juvenile hormone analog, which initiated further embryonic development in diapause eggs [19]. Hunt *et al*. reported suppression of juvenile hormone production in paper wasps with the expression of a hexameric storage protein leading to accumulation of energy storage in diapause conditions [33]. In other insect species heat-shock protein (hsp) expression plays a crucial role in cold survival [17], and another juvenile hormone analog, methoprene, inhibited expression of small hsp in *Drosophila* [34]. In contrast, ecdysteroid and their derivatives, which play roles during multiple embryonic development events, exhibit diapause regulation in *A*. *albopictus* and other mosquitoes when transcriptome analysis of mature oocyte and developing embryos is performed [21,27,32]. Ecdysteroid has also been associated with maintenance of diapause in pharate larvae of gypsy moth, *Lymantaria dispar* [35]. The mechanism of diapause termination in *A*. *albopictus* using pyriproxyfen is unclear. Further studies are required to determine whether pyriproxyfen interferes ecdysteroid hormone or any other metabolic activity by inhibiting hsp synthesis and storage proteins or by activating ERK phosphorylation.

## Conclusion

We demonstrated that pyriproxyfen is not only a diapause terminator in *A*. *albopictus* eggs, but also acts as an ovicide, killing most diapause eggs at 1 ppm. Our study also provides a precise window for effective diapause egg treatment, which could be a novel tool for mosquito management. This suggests that pyriproxyfen application before the onset of diapause-inducing cues may reduce population buildup for the next mosquito season.
